# Management of pregnancy, delivery and breast-feeding in hereditary angioedema: an analysis of 15 pregnancies with conventional treatment approaches and a case of Lanadelumab use

**DOI:** 10.1186/s13023-026-04280-y

**Published:** 2026-02-21

**Authors:** Marine Casanova, Marie-Charlotte Brüggen, Walter Alfred Wuillemin, Christina Weber-Chrysochoou

**Affiliations:** 1 Allergy Unit, Department of Dermatology, University Hospital Zurich, The Circle 59, Zurich, 8058 Switzerland; 2 Faculty of Medicine, University Zürich, Rämistrasse 71, Zurich, 8006 Switzerland; 3 Division of Haematology, Department of Internal Medicine, Cantonal Hospital Lucerne, Spitalstrasse 16, Luzern, 6000 Switzerland

**Keywords:** Breast-feeding, C1 inhibitor deficiency, Hereditary angioedema, Lanadelumab, Pregnancy, Treatment

## Abstract

**Background:**

The course of hereditary angioedema (HAE) during pregnancy varies greatly. Limited treatment options complicate the management of HAE in this period. This study aimed to examine the clinical progression of HAE throughout the perinatal period in relation to management strategies. Additionally, we reported the first documented case of Lanadelumab (Takhzyro) therapy during pregnancy in a patient with HAE.

**Results:**

We performed an observational study at the University Hospital Zurich (a tertiary treatment center), in which we included 7 HAE female HAE patients. We analyzed 15 pregnancies of these women. The average maternal age at delivery was 33 years (range: 21–41), and the average gestational age was 40 + 3/7 weeks. Fourteen deliveries were spontaneous, with one requiring vacuum extraction. There were no miscarriages during the study period. Maternal C1-INH levels averaged 0.09 g/l (range: 0.04–0.16), and functional activity averaged 28% (range: 17–42%). Of the 15 newborns, 8 were HAE-positive. Their mean birth weight was 3170 g (±546), compared to 3618 g (±225) in HAE-negative children. No significant complications or adverse outcomes were observed in either group. Attack frequency during pregnancy increased in 4 cases, decreased in 7, and remained unchanged in 4. Most women (60%) received plasma-derived C1-inhibitor (Berinert) for acute management; 27% used it prophylactically during pregnancy. One patient received combination therapy with Lanadelumab/Takhzyro® and Berinert®, showing good tolerability and effectiveness. The pregnancy proceeded without complications and the child was born healthy at term with normal birth weight.

**Conclusions:**

The variable evolution of HAE symptoms during pregnancy highlights the need for flexible and personalized treatment plans. Plasma-derived C1-inhibitor remains the recommended treatment during pregnancy due to its favorable therapeutic and safety profile. The first documented use of Lanadelumab during pregnancy was well tolerated and linked to positive outcomes for both mother and newborn. Although these findings are promising, further research is necessary to confirm the safety and effectiveness of Lanadelumab in this setting.

## Background

Hereditary Angioedema (HAE) is an autosomal-dominant genetic disease [1] . It is a rare disease affecting approximately 1 in 50’000 individuals, with reported incidence rates ranging from 1:10’000 to 1:150’000 in the general population [2]. The condition causes episodes of cutaneous or submucosal edema, most commonly affecting the upper respiratory and gastrointestinal tracts [3]. For patients, these potentially life-threatening episodes can be extremely distressing. This emphasizes the importance of understanding the underlying pathophysiology of HAE and providing appropriate clinical management.

HAE is a bradykinin-mediated angioedema caused by a deficiency or defect of C1 inhibitor (C1-INH) or other mechanisms [1, 3]. A distinction is made between two types: type 1 (HAE-1) and type 2 (HAE-2). HAE-1 results from a C1-INH deficiency, leading to low antigenic and functional C1-INH levels, whereas HAE-2 is caused by C1-INH dysfunction, with normal (or high) antigenic levels but reduced function [3, 4]. Additionally, HAE with normal C1-INH levels is recognized as a rare subtype associated with various genetic mutations [3]. *C1-INH is a serine protease inhibitor (SERPIN) encoded by the SERPING1 gene* [4]. *A mutation in this gene results in a deficiency or defect of C1-INH, allowing factor XII and plasma kallikrein to be activated*. This activation triggers the release of bradykinin, which modulates vascular permeability and leads to episodes of edema [3, 5].

While bradykinin release is responsible for swelling episodes, common triggers of HAE attacks are emotional stress, mechanical trauma, infections, and hormonal changes [6]. Elevated estrogen levels from menstruation, pregnancy, or estrogen-containing contraceptives are well-documented hormonal triggers of swelling episodes in women with HAE [5]. Pregnancy, therefore, has a highly variable and unpredictable impact on HAE: it can lead to symptom worsening, improvement, or no change in symptoms [7]. During pregnancy, angioedema episodes most commonly affect the abdomen, followed by the limbs. The increased incidence of abdominal episodes may be explained by fetal movement and mechanical stress resulting from the progressive growth of the fetus and uterus [8], although this was the only description back in 1972, it was never confirmed. Submucosal edema of the airways are uncommon in pregnancy but potentially fatal and must be treated as a medical emergency [9].

Given the variable impact of pregnancy and other triggers on HAE attacks, effective long-term management strategies are essential. Long-term prophylactic treatment (LTP) of HAE is recommended with plasma-derived C1-INH (pdC1-INH), such as Berinert. This treatment is effective and well tolerated, offering improved quality of life in patients with frequent HAE attacks. Berinert is shown to be safe during pregnancy and breast-feeding [3]. Alternatively, Lanadelumab can be used for LTP, but it is not yet recommended during pregnancy and breast-feeding. Lanadelumab is a monoclonal antibody that targets plasma kallikrein and has a favorable safety profile, making it an effective prophylactic treatment [3]. Kallikrein inhibitors such as Berotralstat also provide a convenient oral prophylactic option but are also not recommended during pregnancy and lactation [3]. For the long-term prevention of HAE-1/-2, attenuated androgens have long been utilized [3]. Androgen derivatives have been shown to be effective in HAE-1/-2 prophylaxis, and oral administration makes them more accessible [3]. However, androgens must be used with caution, particularly in view of their androgenic and anabolic effects, medication interactions, and contraindications [7, 10]. For long-term prophylaxis, antifibrinolytics, such as tranexamic acid, are no longer recommended. Although there is lack of evidence supporting their effectiveness, certain patients might benefit from them [8, 11].

In some cases, additional on-demand treatment or only on-demand treatment is required for HAE patients, especially if they experience laryngeal angioedema. First-line on-demand therapies include intravenous C1-INH, Icatibant, and Ecallantide. Icatibant is a self-administered bradykinin B2 receptor antagonist that mediates vasodilatation and enhances capillary permeability [12]. Because of the risk of hypersensitivity reactions, Ecallantide, a kallikrein inhibitor, must be given subcutaneously by a healthcare professional [13–15].

While numerous treatment options exist for the prevention and acute management of angioedema attacks in patients, options for pregnant women are much more limited. Due to its longstanding safety and efficacy, pd-C1-INH such as Berinert is the preferred treatment for all therapy modalities during pregnancy. Berinert can be administered subcutaneously or intravenously. The method of administration depends on the treatment purpose (acute vs. prophylactic), patient factors, dosing convenience, and both physician and patient preferences. Since there is considerably less experience with alternative treatment options, we aimed to investigate a potential substitute for intravenous pdC1-INH during pregnancy. We compared various prophylactic and acute treatment regimens in women with HAE before, during, and after pregnancy, alongside the progression of angioedema attacks across these periods. Among the cases observed, Lanadelumab (Takhzyro) was used together with pdC1-INH (Berinert) for acute management. As there is currently no published experience with using Lanadelumab during pregnancy, and its use is off-label and not recommended, our goal is to report the clinical course of a pregnancy in an HAE patient treated with Lanadelumab to contribute to a better understanding of alternative therapeutic options.

## Methods

This observational study was conducted at the University Hospital of Zurich, Switzerland, in collaboration with the Lucerne Cantonal Hospital and the Bethesda Hospital Basel. Our study was approved by the cantonal ethics committee (BASEC Nr. 2023–01329). All participants gave their written informed consent. Inclusion criteria were a confirmed diagnosis of HAE and at least one pregnancy completed during the study period. We investigated 15 pregnancies from 8 women with HAE Type-1 from 1991 to 2025. Data were collected and entered in a clinical data base. This included information for the periods before pregnancy, during pregnancy, labor, and the postpartum period.

The following data were obtained via patient diaries, medical history and interviews:

- Patient demographics.
- Attack rate before, during and after pregnancy as well as localization of HAE attacks.
- HAE therapy prior to pregnancy, during pregnancy and during postpartum period.
- Therapy during labor as well as pregnancy outcome including delivery type and timing.
- Characteristics of newborn children, HAE-Status, C1-INH values, C1-INH-function, C4 levels and if available genetics.
- Maternal levels of C1-INH, C1-INH function and C4 levels.

Statistical analysis was performed using R studio (program version 4.4.3). Data were analyzed descriptively and provided in the form of tables and figures.

## Results

### Demographics and pregnancy details

A total of 15 pregnancies from 8 women were included in the analysis (Table 1). Seven women experienced two pregnancies, while one had a single pregnancy. The average maternal age at the time of data collection was 47 years (range, 28–64 years). All patients were diagnosed with type 1 HAE. Maternal age at the time of delivery ranged from 21 to 41 years, with a mean age of 33 years. The average gestational duration was 40 + 3/7 weeks (range, 38 + 1/7 - 41 + 2/7 GW). Of the 15 pregnancies, 14 resulted in spontaneous vaginal deliveries, and one was assisted by vacuum extraction; no cesarean sections were reported. Two patients had a history of spontaneous abortion, though no miscarriages occurred during the observed pregnancies.

**Table 1:** Demographic data

Patients (n = 8)		Reference range
Gestational age (mean, range)	33 (21–41) years	
Gestational week (mean, range)	40 3/7 (38 1/7–41 2/7) GW	
C1-INH quantitative (mean, range)	0.09 (0.04–0.16) g/l	0.20–0.34 g/l
C1-INH functional (mean, range)	28 (17–42) %	>70%
C4 (mean, range)	0.08 (0.07–0.12) g/l	0.10–0.40 g/l
**Type of birth**		
Spontaneous birth	14	
Vacuum birth	1	
**Children (n = 15)**		
Children without HAE	7	
Children with HAE	8	
Birth weight (mean, range)	3561 (2880–4700) g	
C1-INH quantitative (mean, range)	0.1 (0.04–0.18) g/l	0.21–0.38 g/l
C1-INH functional (mean, range)	39 (13–63) %	>70%
C4 (mean, range)	0.09 (0.07–0.12) g/l	0.07–0.23 g/l
**Genetics (n = 2)**		
SERPING1-variant	c1418T > G p.(Val473Gly) heterozygote	

C1-INH, C1 esterase inhibitor; C4, Complement factor 4; HAE, Hereditary Angioedema

Overall, 15 children were born (Table 1). The average birth weight was reported as 3561 g (range, 2880–4700 g). Among these, 8 out of the 15 children (53%) were diagnosed with HAE, while 7 showed no signs of the disease. C1-INH concentrations ranged from 0.04 to 0.16 g/l, with a mean of 0.09 g/l. The mean C1-INH function was 28% (range, 17–42%), and the mean C4 level was 0.08 g/l (range, 0.04–0.12 g/l). Genetic analysis was performed on two neonates (siblings) using umbilical cord blood samples collected at birth, revealing a heterozygous c.1418T > G p. (Val473Gly) mutation in the SERPING1 gene, which was already known from the mother.

### Characteristics of children with and without HAE

On average, children diagnosed with HAE weighed 3170 grams (SD ±546 g), while children without HAE had a mean birth weight of 3618 grams (SD ±225 g) (Table 2). This difference was not statistically significant (mean difference − 448 g, *p* = 0.066). The average gestational age at delivery was similar between the two groups, with HAE-positive children delivered at 40 + 1 weeks of gestation (SD ±0.9 weeks) and HAE-negative children at 40 + 5 weeks (SD ±0.7 weeks). Regarding swelling episodes during pregnancy, most pregnant women reported no such events, regardless of the child’s HAE status. Among women carrying HAE-positive children, 25% received prophylactic treatment with Berinert, and 50% had access to Berinert for acute symptom management. In pregnancies resulting in HAE-negative children, 28.6% of mothers were given Berinert prophylactically, while 71.4% had access to Berinert for emergency treatment if needed. However, a significant proportion of pregnant women did not receive specific prophylactic treatment during pregnancy, with 62.5% in the HAE-positive group and 71.4% in the HAE-negative group.

**Table 2:** Comparison of children with and without HAE

	With HAE (*n* = 8)	Without HAE (*n* = 7)
Birth weight (mean ± SD)	3170 ± 546 g	3618 ± 225 g
Gestational week (mean ± SD)	40 + 1 GW ±0.9 Week	40 + 5 GW ±0.7 Week
Frequency of swelling during pregnancy	None: 62.5%	None: 60%
	Rarely (less than once a month): 12.5%	Rarely (less than once a month): 0%
	Once per week to once per month: 0%	Once per week to once per month: 0%
	1–2 times per week: 25%	1–2 times per week: 40%
Prophylaxis during pregnancy	Berinert: 25%	Berinert: 28.6%
	None: 62.5%	None: 71.4%
	Berinert/Takhzyro*: 12.5%	Berinert/Takhzyro*: 0%
Acute treatment during pregnancy	Berinert: 50%	Berinert: 71.4%
	None: 50%	None: 28.6%

* Patient received Berinert i.v. every 2 weeks during the first trimester. From the second trimester onwards, Takhzyro was administered every month and Berinert was used as an acute treatment

### HAE symptom evolution

**Fig. 1:**
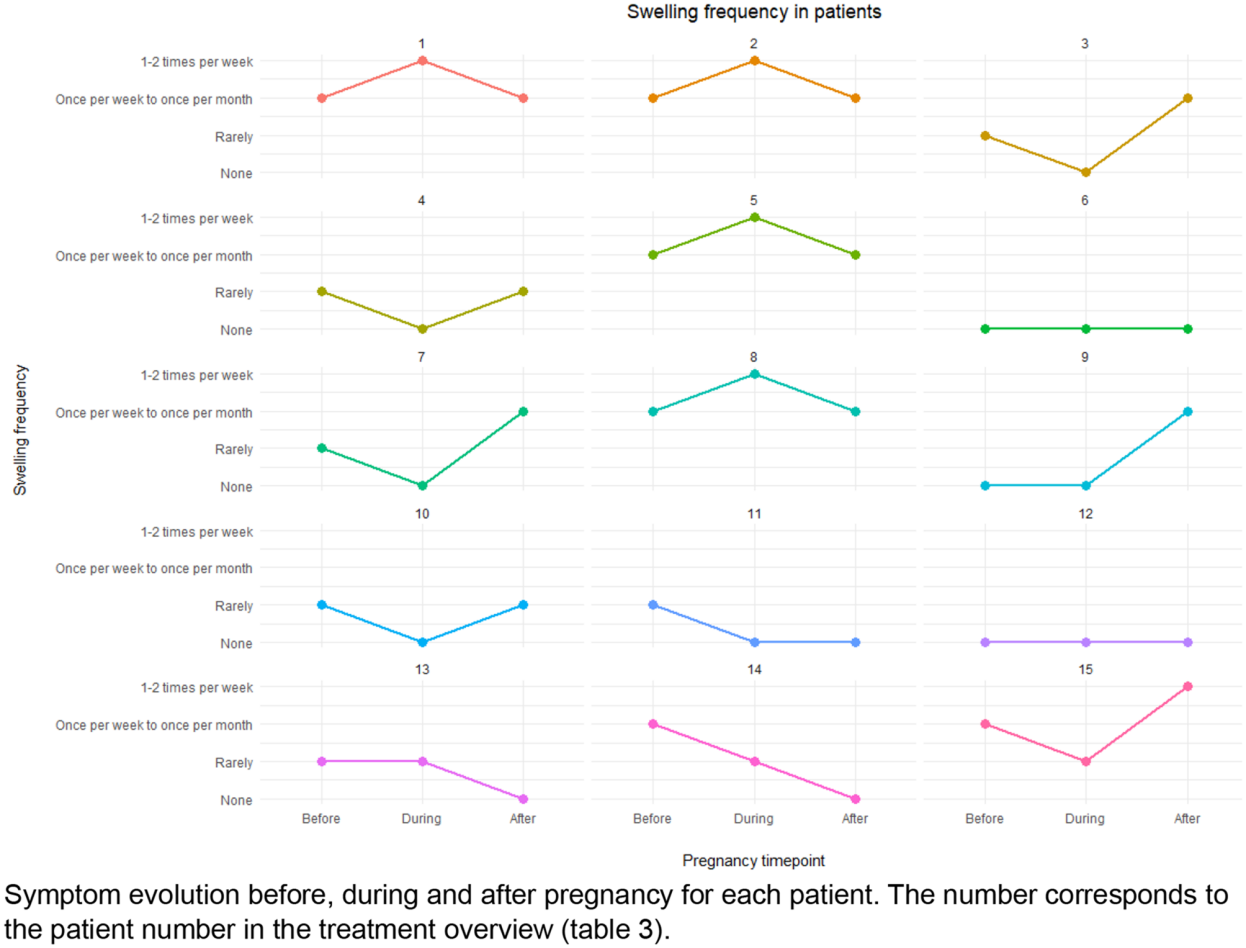
Symptom evolution before, during and after pregnancy for each patient. The number corresponds to the patient number in the treatment overview (Table 3)

**Fig. 2:**
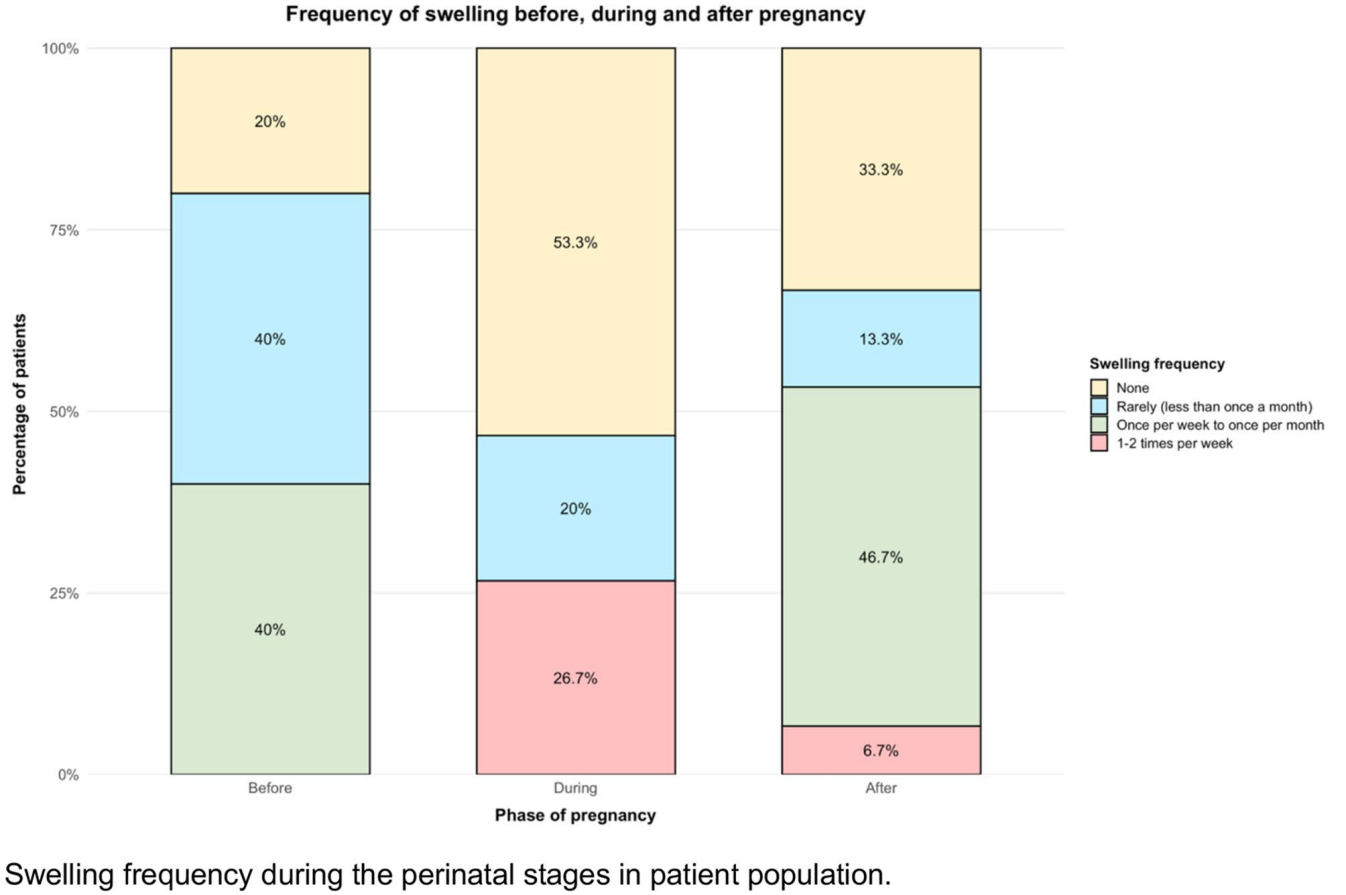
Swelling frequency during the perinatal stages in patient population

HAE symptom patterns varied among individuals during the perinatal period (Fig. 1). During pregnancy, four patients reported an increase in the frequency of HAE attacks, seven patients reported a decrease, and four patients experienced no change (a limitation of the study is that there are no detailed numbers). The proportion of pregnant women reporting no swelling increased from 20% pre-pregnancy to 53.3% during pregnancy, then decreased to 33.3% in the postpartum period (Fig. 2). A small subgroup of women (26.7%) experienced an increase in attack frequency to 1–2 episodes per week during pregnancy, which later declined to 6.7% after delivery.

### Therapeutical management

**Fig. 3:**
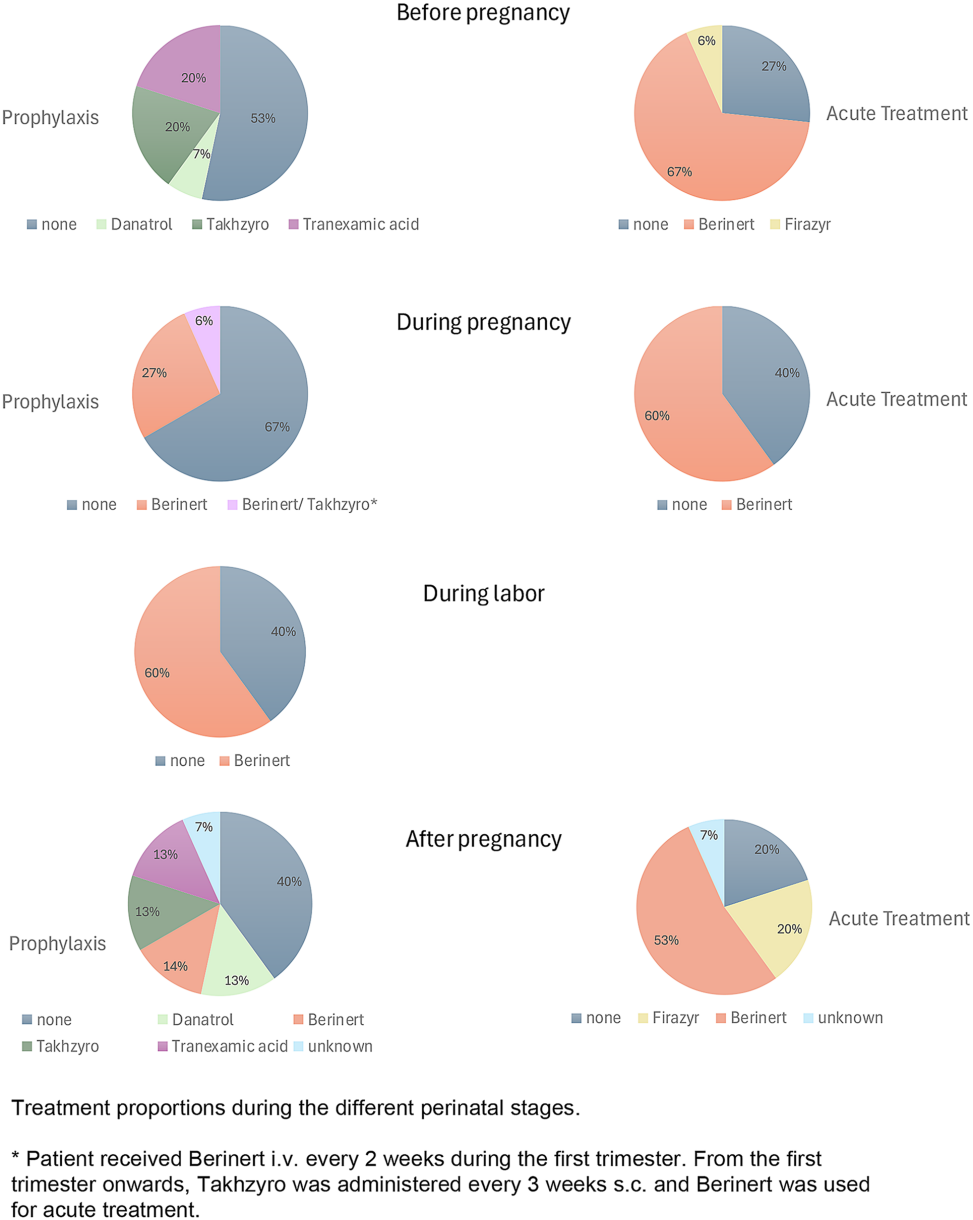
Treatment proportions during the different perinatal stages. * patient received Berinert i.v. every 2 weeks during the first trimester. From the first trimester onwards, Takhzyro was administered every 3 weeks s.c. and Berinert was used for acute treatment

Therapeutic management was tailored to the specific stages of the perinatal period, with personalized treatment plans for each patient (Table 3). Before pregnancy, 53% of patients received no preventive treatment, 20% used Lanadelumab (Takhzyro), 20% used tranexamic acid, and 7% used androgens (Danatrol) (Fig. 3). Nearly all patients had access to acute treatment before pregnancy, most commonly plasma-derived C1-inhibitor (Berinert) (67%) and Icatibant (Firazyr) (6%), while 27% lacked an acute treatment option during this period.

**Table 3:** Therapy overview

	Before pregnancy		During pregnancy		During labor	After pregnancy	
Patient number	Prophylaxis	Acute treatment	Prophylaxis	Acute treatment	Treatment	Prophylaxis	Acute treatment
11	none	none	none	none	none	none	none
12	none	none	none	none	none	none	Firazyr
9	none	none	none	none	none	none	Firazyr
4	none	Berinert	none	none	none	none	none
7	none	Berinert	none	none	1000 IE Berinert	Danatrol	Berinert
5	none	Berinert	Berinert	Berinert	1500 IE Berinert before and after	Berinert	Berinert
13	none	Berinert	Berinert	Berinert	none	Berinert	Berinert
10	none	Firazyr	none	Berinert	1500 IE Berinert	none	Firazyr
8	Danatrol	Berinert	Berinert	Berinert	1000 IE Berinert	Danatrol	Berinert
15	Takhzyro	none	none	Berinert	1500 IE Berinert	unknown	unknown
6	Takhzyro	Berinert	Berinert	Berinert	1500 IE Berinert	Takhzyro	Berinert
**14**	**Takhzyro**	**Berinert**	Berinert/Takhzyro*	**Berinert**	**1500 IE Berinert before and after**	**Takhzyro**	**Berinert**
3	Tranexamic acid	Berinert	none	none	none	none	none
1	Tranexamic acid	Berinert	none	Berinert	1500 IE Berinert before and after	Tranexamic acid	Berinert
2	Tranexamic acid	Berinert	none	Berinert	1500 IE Berinert before and after	Tranexamic acid	Berinert

* Patient received Berinert i.v. every 2 weeks during the first trimester. From the first trimester onwards, Takhzyro was administered every 3 weeks s.c. and Berinert was used for acute treatment

During pregnancy, 60% of patients were treated acutely with plasma-derived C1-inhibitor (Berinert), and 27% received it prophylactically. C1-inhibitor (Berinert) was primarily administered intravenously, as most patients declined subcutaneous administration due to the larger volume needed. Subcutaneous C1-inhibitor (Berinert) was administered in only one case. One patient received a combination prophylactic regimen with Lanadelumab (Takhzyro) and plasma-derived C1-inhibitor (Berinert), along with plasma-derived C1-inhibitor (Berinert) for acute attacks—a case that will be discussed in more detail below. At the time of delivery, 60% of patients received plasma-derived C1-inhibitor (Berinert), while 40% did not require any therapy.

During the postpartum period, 40% of patients received no prophylaxis, while 14% continued using plasma-derived C1-inhibitor (Berinert). The synthetic androgen Danazol, Lanadelumab (Takhzyro), and tranexamic acid were each used as prophylaxis in 13% of patients. For acute treatment after childbirth, 53% used plasma-derived C1-inhibitor (Berinert), 20% received Icatibant (Firazyr), and 20% required no acute intervention. Only one mother was not breastfeeding, the others were breastfeeding between 2 and 24 months after birth.

### Lanadelumab (Takhzyro) during pregnancy

**Fig. 4:**
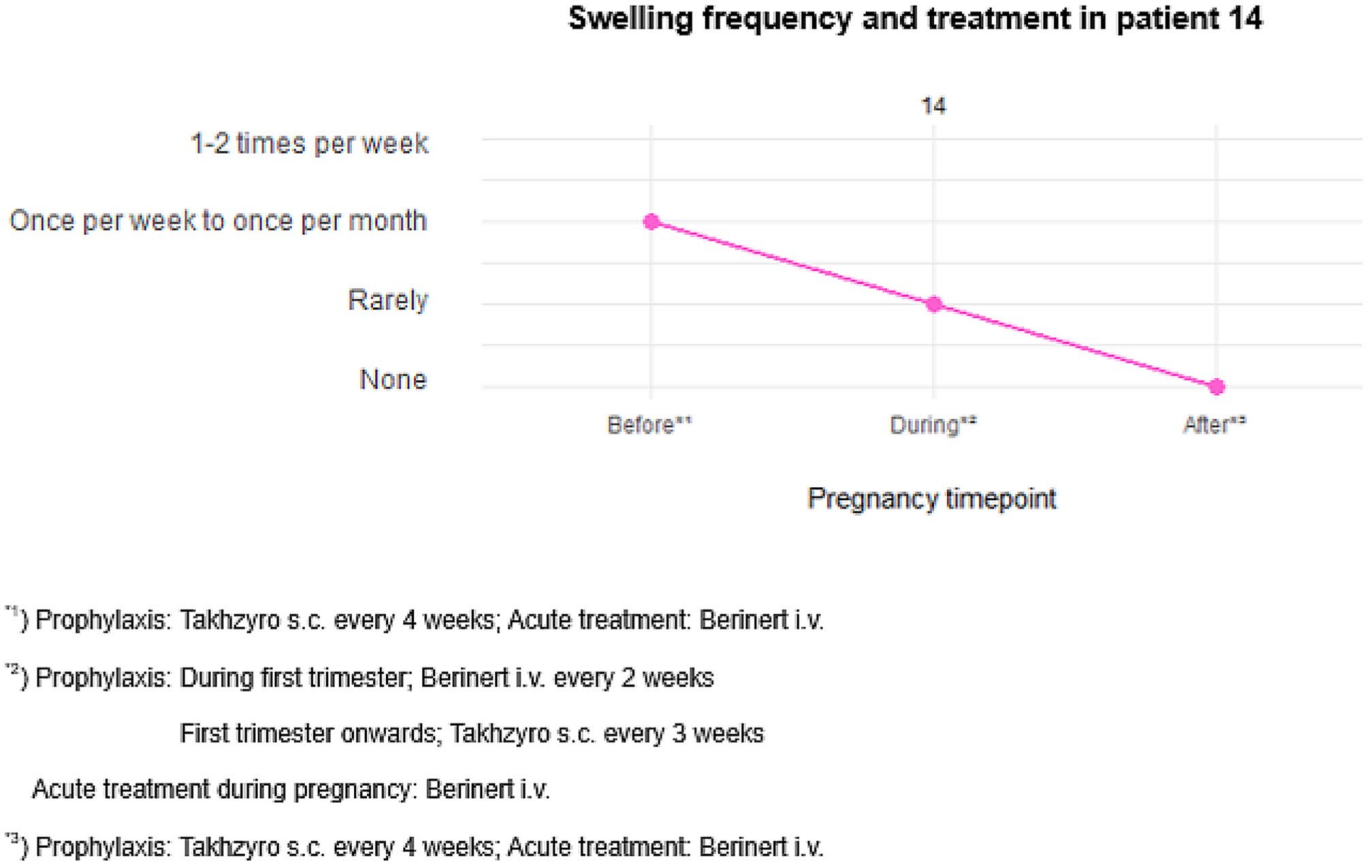
*1) Prophylaxis: Takhzyro s.c. every 4 weeks; acute treatment: Berinert i.v. *2) Prophylaxis: during first trimester: Berinert i.v. every 2 weeks, first trimester onwards: Takhzyro s.c. every 3 weeks; Acute treatment during pregnancy: Berinert i.v. *3) Prophylaxis: Takhzyro s.c. every 4 weeks; acute treatment: Berinert i.v

This case describes a 38-year-old woman with HAE type 1 and a positive family history (Table 4, Fig. 4). Before her first pregnancy, she experienced monthly angioedema attacks, which decreased during pregnancy after she received intensified prophylactic therapy with intravenous plasma-derived C1-inhibitor (Berinert) twice weekly. No treatment was needed during labor and postpartum, and no further swelling occurred.

**Table 4:** Pregnancy under Takhzyro/Berinert therapy

Parameter	
HAE status	Positive
Birth weight (g)	4000
Gestational week	40 + 5
Frequency of swelling	Before pregnancy: Once per week to once per month During pregnancy: Rarely (less than once a month) After pregnancy: None
Prophylaxis before pregnancy	Takhzyro s.c. every 4 weeks
Acute treatment before pregnancy	Berinert i.v.
Prophylaxis during pregnancy	During first trimester: Berinert i.v. every 2 weeks First trimester onwards: Takhzyro s.c. every 3 weeks
Acute treatment during pregnancy	Berinert i.v.
Treatment during labor	1500 IE Berinert before and after labor
Prophylaxis after pregnancy	Takhzyro s.c. every 4 weeks
Acute treatment after pregnancy	Berinert i.v.

HAE, hereditary angioedema

Prior to her second pregnancy, the patient had been on prophylactic therapy with Lanadelumab (Takhzyro) for two years, initially administered every two weeks and later extended to four-week intervals, with intravenous Berinert available for acute treatment. During this time, she experienced an HAE attack approximately every three to four months. After becoming pregnant, she was advised to discontinue Lanadelumab and switch to plasma-derived C1-inhibitor (Berinert) every two weeks. Attacks recurred, and she had to increase the frequency of administration to 2–3 times a week. This became unbearable for her, and she resumed combination therapy with subcutaneous Lanadelumab (Takhzyro) every three weeks and on-demand plasma-derived C1-inhibitor (Berinert) (Table 4). She informed her attending physician that she would continue this treatment at her own risk. Because she already had enough medication supply, stopping the prescription had no effect. The situation was assessed by the institutional ethics board, which concluded that there were insufficient reasons to prevent her from doing so through official measures.

The frequency of attacks remained low and was even lower than during the first pregnancy, primarily affecting the extremities and abdomen. Lanadelumab (Takhzyro) prophylaxis was well tolerated throughout the pregnancy and breastfeeding, which proceeded without complications. A total of 1500 IU of plasma-derived C1-inhibitor (Berinert) was administered intravenously before and after labor. The delivery was spontaneous at 40 weeks and 6 days of gestation; the newborn was healthy with a normal birth weight of 4000 grams, the mother was breastfeeding. At age two, the child was also diagnosed with HAE but otherwise healthy.

## Discussion

### Principal findings

The study examined the course of HAE attacks during the perinatal period in 15 pregnancies. The results reveal a high variability in HAE symptom progression, with attack frequency either increasing, decreasing, or staying the same throughout pregnancy. There were no abortions, fetal or maternal drug-related problems, and pregnancies proceeded without complications. Newborn birth weights were within expected ranges, and no adverse neonatal outcomes were observed. Furthermore, the HAE status of the offspring did not seem to affect maternal attack frequency. We also observed effective management of HAE symptoms during pregnancy with plasma-derived C1-inhibitor (Berinert). Despite limited treatment options for HAE during pregnancy, plasma-derived C1-inhibitor (Berinert) was shown to be a safe and effective treatment for both prevention and treatment of attacks. A positive outcome for mother and baby was seen in one case involving combination therapy with Lanadelumab (Takhzyro) and plasma-derived C1-inhibitor (Berinert), indicating a potential alternative therapy during pregnancy. It should be noted that this treatment was used in only one of the pregnancies studied. While this indicates that Lanadelumab may be a safe option during pregnancy, it is not approved for this use. Additional data are urgently needed before any conclusions can be drawn about the safety of Lanadelumab during pregnancy.

### Results in the context of what is known

The variability of HAE symptoms during pregnancy, as observed in this study, aligns with findings from previous research. Several studies have reported similar fluctuations in symptom severity, highlighting the complex progression of HAE during pregnancy [16–19]. However, some studies have documented an increased attack rate during pregnancy [16], indicating that pregnancy may worsen HAE symptoms for certain patients. These contrasting findings could be due to differences in study populations, hormonal effects, or treatment strategies.

In the 15 pregnancies observed in our study, newborn weight was within the expected ranges. Moreover, HAE status in children didn’t seem to influence the frequency of HAE attacks. These findings are in line with studies showing that the child’s HAE status does not affect the course of symptoms during pregnancy [19].

### Clinical implications

The variability of HAE symptoms during pregnancy underscores the need for personalized management of HAE in pregnant women. The variety of therapeutic regimens used across patients and perinatal stages highlights that no single approach works for everyone. Due to the changing nature of HAE symptom expression during and after pregnancy, flexible and closely monitored treatment plans should be considered.

In the case presented, Lanadelumab, started in the second trimester, was well tolerated and linked with a reduced frequency of attacks, suggesting that it may be safe and effective during pregnancy. Although Lanadelumab is approved for LTP therapy of HAE attacks in adults and has shown a significant decrease in attack frequencies in several studies, it is currently not recommended for use during pregnancy due to limited clinical data [3, 20]. Lanadelumab offers a convenient alternative to intravenous options because it requires a subcutaneous dose every two to four weeks, which may improve adherence and quality of life, especially during pregnancy. Such treatment options could help improve perinatal management of HAE.

### Research implications

This study offers valuable insights into HAE treatment during pregnancy. However, the reasons for the variability in symptom progression among pregnant patients remain unclear. Hormonal fluctuations, especially rising estrogen levels, are believed to significantly influence disease activity [21]. Trimester-specific hormonal changes [19, 22], known triggers such as menstruation or physical trauma [18], and the discontinuation of prophylactic therapy upon conception [16, 18] may also contribute. Mechanical stressors, like uterine and abdominal wall expansion, could further worsen abdominal attacks [23]. Future research should seek to clarify these mechanisms to enhance understanding of HAE’s pathophysiology in pregnancy. Additionally, before routinely recommending newer prophylactic treatments, such as Lanadelumab (Takhzyro), in pregnant women, prospective studies are necessary to assess their safety and efficacy. Nonetheless, the availability of these therapeutic options offers hope for better care and increased treatment choices for patients with HAE during pregnancy.

### Strengths and limitations

The strength of this study is the detailed documentation of how HAE attack symptoms evolve during the perinatal period, along with the associated therapeutic management across these different phases. It also presents the first documented case of pregnancy managed with Lanadelumab (Takhzyro), highlighting a possible alternative treatment during pregnancy. Despite limitations such as a small sample size and the observational nature of the study, these findings offer valuable insights into managing HAE in pregnant individuals.

## Conclusions

In conclusion, this study highlights the variability in HAE symptom progression during pregnancy. The influence of hormonal changes on HAE symptoms, particularly during pregnancy, remains insufficiently understood. The fluctuations of HAE symptoms during pregnancy observed in our cohort highlights this gap and indicates that further research is needed to better understand how hormonal factors affect disease activity during pregnancy. Our study provides initial data addressing this underexplored area and may serve as a foundation for future investigations. Additionally, the study also supports the effectiveness of personalized treatment strategies. Plasma-derived C1-inhibitor (Berinert) showed a good safety and efficacy profile for both emergency and preventive use, aligning with previous clinical data. The successful application of Lanadelumab (Takhzyro) in one case suggests it could be a promising option during pregnancy, though more research is necessary to confirm its safety. Overall, these findings emphasize the importance of tailored treatment approaches and encourage further research into safe and effective options for managing pregnancy-related HAE.

## Data Availability

The datasets used and/or analysed during the current study are available from the corresponding author on reasonable request.
